# Comparison of Diagnostic Accuracy of Radiation Dose-Equivalent Radiography, Multidetector Computed Tomography and Cone Beam Computed Tomography for Fractures of Adult Cadaveric Wrists

**DOI:** 10.1371/journal.pone.0164859

**Published:** 2016-10-27

**Authors:** Jakob Neubauer, Matthias Benndorf, Carolin Reidelbach, Tobias Krauß, Florian Lampert, Horst Zajonc, Elmar Kotter, Mathias Langer, Martin Fiebich, Sebastian M. Goerke

**Affiliations:** 1 Department of Radiology, University Medical Center Freiburg, Freiburg, Germany; 2 Department of Plastic and Hand Surgery, University Medical Center Freiburg, Freiburg, Germany; 3 Department of Medical Physics and Radiation Protection, University of Applied Sciences Gießen, Gießen, Germany; 4 Department of Radiology, Ortenau Klinikum Offenburg-Gengenbach, Offenburg, Germany; Northwestern University Feinberg School of Medicine, UNITED STATES

## Abstract

**Purpose:**

To compare the diagnostic accuracy of radiography, to radiography equivalent dose multidetector computed tomography (RED-MDCT) and to radiography equivalent dose cone beam computed tomography (RED-CBCT) for wrist fractures.

**Methods:**

As study subjects we obtained 10 cadaveric human hands from body donors. Distal radius, distal ulna and carpal bones (n = 100) were artificially fractured in random order in a controlled experimental setting. We performed radiation dose equivalent radiography (settings as in standard clinical care), RED-MDCT in a 320 row MDCT with single shot mode and RED-CBCT in a device dedicated to musculoskeletal imaging. Three raters independently evaluated the resulting images for fractures and the level of confidence for each finding. Gold standard was evaluated by consensus reading of a high-dose MDCT.

**Results:**

Pooled sensitivity was higher in RED-MDCT with 0.89 and RED-MDCT with 0.81 compared to radiography with 0.54 (P = < .004). No significant differences were detected concerning the modalities’ specificities (with values between P = .98). Raters' confidence was higher in RED-MDCT and RED-CBCT compared to radiography (P < .001).

**Conclusion:**

The diagnostic accuracy of RED-MDCT and RED-CBCT for wrist fractures proved to be similar and in some parts even higher compared to radiography. Readers are more confident in their reporting with the cross sectional modalities. Dose equivalent cross sectional computed tomography of the wrist could replace plain radiography for fracture diagnosis in the long run.

## Introduction

The fracture of the distal forearm is one of the most common types of fractures. Together with fractures of the carpus they account for over 50% of all fractures in the upper extremity [1]. Radiography is recommended if a fracture of the distal forearm or the carpus is suspected after wrist trauma [2]. Computed tomography (CT), however, has been shown to perform superior to radiography for diagnosis of these fractures. Accordingly CT has been found to have a higher sensitivity for fractures of the carpus [3,4] and to be more accurate in evaluation of displacement and joint involvement for fractures of the distal radius [5–7]. The main drawback of CT is the higher amount of radiation.

However, it has been shown that multidetector computed tomography (MDCT) imaging of the wrist is also possible in low dose settings [8]. In addition to MDCT, cone beam computed tomography (CBCT) has been described as a potentially low dose cross sectional imaging modality in musculoskeletal radiology [9,10]. CBCT, which is already established in maxillofacial imaging [11], is regarded an emerging imaging modality in musculoskeletal extremity imaging [9,10,12–15]. CBCT can provide higher spatial resolution but performs inferior in terms of contrast resolution and amount of imaging artifacts when compared to MDCT [16,17].

Given the superior diagnostic performance of computed tomography for carpal and distal forearm fractures, the aim of our study is to examine whether the applied radiation dose of MDCT and CBCT can be reduced to that of plain radiographs while maintaining the high diagnostic accuracy. If so, diagnostic management of patients with suspected forearm fracture could, in the long run, be altered and the initial evaluation with plain radiographs be skipped. Our hypothesis is that at same dose levels MDCT and CBCT can outperform radiography regarding the diagnostic accuracy of wrist fractures. Therefore we compared the diagnostic accuracy of radiography, to radiography equivalent dose multidetector computed tomography (RED-MDCT) in a 320 row MDCT with single shot mode and to radiography equivalent dose cone beam computed tomography (RED-CBCT) in a device dedicated to musculoskeletal imaging for wrist and carpal fractures.

## Materials and Methods

The Ethics Commission of the University of Freiburg approved this prospective study. All hands were obtained from volunteer body donors. Prior to their death the body donors had provided a written informed consent to donate their body for educational and scientific purposes. This written informed consent is recorded in our institutions Department of Anatomy. The Ethics Commission of the University of Freiburg approved this consent procedure.

### Cadaver specimens

A total of 10 formaldehyde-fixed cadaver specimens are obtained from body donors of our institutions Department of Anatomy. The specimens all include the distal forearm (radius and ulna) and the carpus, resulting in a sample size of 100 bones. Different trauma simulations are carried out on randomly selected bones of the distal forearm and the carpus under a standardized environment in an operating room with fluoroscopy. For trauma simulation each bone is treated separately; fracture patterns involving multiple bones are not established. The decision whether to fracture a bone or not is based on a dice roll with 1 leading to fracturing and 2–6 accounting for no fracture. Trauma simulations are induced with a dorsopalmar compression force via hammer or gouge of circa 500 N. During the simulations the specimens are watered constantly. After the simulations all skin incisions are closed by skin sutures and the specimens are kept in a water bath to avoid emphysema. The simulations are performed by a senior hand surgeon with 20 years experience and a senior resident of surgery with 4 years of experience and documented simultaneously by an assistant.

### Determination of dose

**Fig 1:**
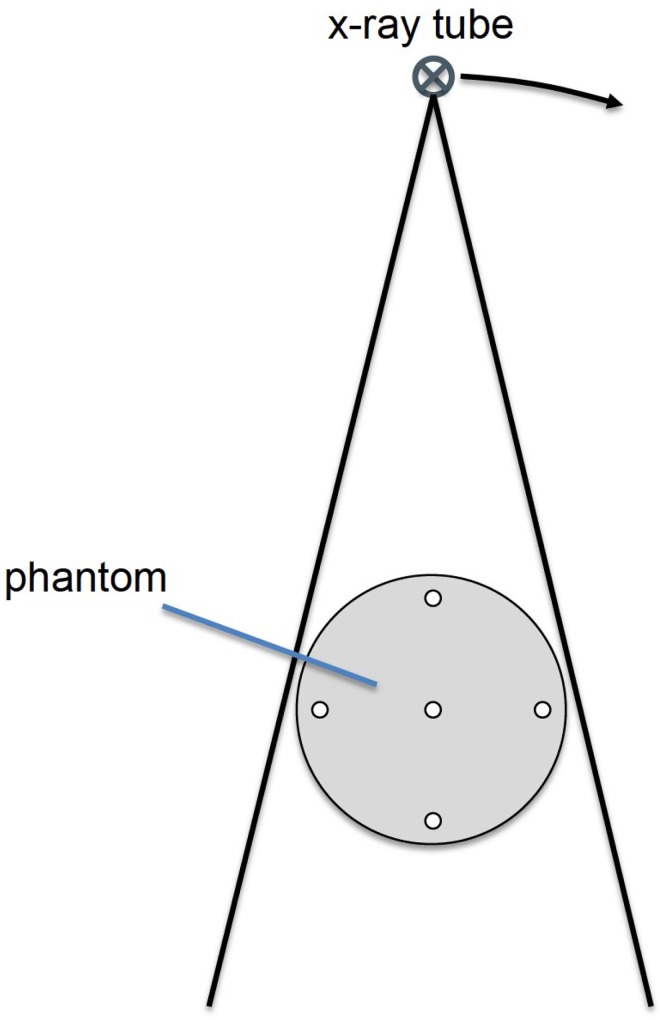
Setup of calibration and validation for Monte Carlos system and experiment. Dose was measured at the five holes with a pin-point chamber in the center of the phantom.

The dose is determined using GMctdospp, a validated Monte Carlo dose calculation system [17,18]. Therefore, the different imaging modalities are modeled into the simulation and a test phantom is used for calibration and for measurements in the simulation and in an experiment (Fig 1). In this model the positions of the tube, of the additional filtration and of the object are used corresponding to the real setup. The energy spectrum used in the simulation corresponds to the kVp settings and the used inherent filtration. The irradiated field in the simulations corresponds to the field used in real life. The object is modeled with the same material and the same geometric properties as in real life. The dose is measured at five different locations in the 16 cm CTDI phantom in radiography, MDCT and CBCT. The same geometry is used in the Monte Carlo simulations and corrected by the measurement data achieving a calibration. In the simulation model a voxel phantom of a lower arm is used. This model is part of the validated voxel model provided by the ICRP [19]. In this model all relevant structures are segmented and can be used in Monte Carlo simulations to calculate organ doses or absorbed doses. In this simulation the sum of all energy doses to all organs is used for comparison of the dose, because in the examined volume there are no more radiosensitive structures. Using this methodology the exposed volume is taken adequately into account.

Using the Monte Carlo simulation the total energy dose for the standard settings of the radiography system is determined initially. For MDCT and CBCT the Monte Carlo simulation is used to find imaging parameters that lead to approximately the same total energy dose.

### Imaging protocols

Radiography (Digital imaging plate system PCR Eleva, Philips, Amsterdam, Netherlands) of the wrist is performed dorsopalmar with 50 kVp/ 2 mAs and lateral with 50 kVp/ 2.5 mAs, resulting in a radiation dose of 2.5 ±0.09 mGy.

RED-MDCT (AquilionOne, Toshiba, Otawara-shi, Japan) is performed in a 180-degree rotation volume mode without pitch (single shot). Settings for kVp and mAs were adjustable stepwise. Therefore we adjust these settings to meet the radiation dose of the radiography as closely as possible without exceeding it. These settings are 100 kVp and 7 mAs, resulting in a radiation dose of 2.31 ±0.05 mGy. The FOV is 16 x 16 x 12.8 cm. Axial images are reconstructed with a matrix of 512 x 512, a slice thickness and sparing of 0.2 mm. The pixel size in the axial plane is 0.3 mm. The images are reconstructed with a bone kernel (FC30).

RED-CBCT (Verity; Planmed, Helsinki, Finland) is performed in a 210-degree rotation mode. Settings for kVp and mAs are adjustable stepwise. Therefore we adjust these settings to meet the radiation dose of the radiography as closely as possible without exceeding it. These settings are 84 kVp and 14,4 mAs, resulting in a radiation dose of 2.17 ±0.05 mGy. The FOV is 16 x 16 x 13 cm. Axial images are reconstructed with a matrix of 801 x 801, a slice thickness and sparing of 0.2 mm. The pixel size in the axial plane is 0.2 mm. The images are reconstructed with a bone kernel (Hamming).

Gold standard imaging is performed in the MDCT with spiral mode at a pitch factor of 0.641, 120 kV and 150 mAs (AquilionOne, Toshiba, Otawara-shi, Japan). This high dose protocol is chosen to provide the best image quality possible. The FOV is 16 x 16 x 12.8 cm. Axial images are reconstructed with a matrix of 512 x 512, a slice thickness and sparing of 0.2 mm. The pixel size in the axial plane is 0.3 mm. The images are reconstructed with a bone kernel (FC30). All images are sent to a picture archiving and communication system (PACS, AGFA Impax 6, Agfa, Mortsel, Belgium).

### Qualitative and quantitative image analysis

A radiologist with 3 years experience (rater 1), a radiologist with 5 years experience (rater 2) and a radiologist with 7 years experience (rater 3) evaluate the images independently in a PACS, window levels in CT are initially set to L/W 350/2000. The raters are free to change window settings and to perform multiplanar reconstructions. Evaluation takes place on workstations with standardized displays (RadiForce RX220; EIZO Corp, Hakusan, Ishikawa, Japan), which are calibrated according to DICOM [20]. Imaging conditions are kept constant. The raters are blinded towards the CT modalities (MDCT versus CBCT). All information in the DICOM files that could make the readers identify the modalities is deleted prior to the presentation. Blinding towards radiography is not possible due to obviously different image appearance. The raters are asked to evaluate the given images for fractures. Also, all fragments of a fracture should be counted. Raters score the certainty of every finding on a 5-point Likert Scale with 1 (= very high certainty), 2 (= high certainty), 3 (= moderate certainty), 4 (= low certainty) and 5 (= very low certainty). The raters are informed that each bone is to be analyzed separately without considering fracture patterns. The equivalent images of the other modality are presented to the readers in different randomized order after 4 weeks, to avoid recall bias. In the first round only radiography images are presented. In the second round 5 RED-MDCT and 5 RED-CBCT scans are presented. In the third round the equivalent images of their CT counterpart modality are presented. The gold standard is evaluated via consensus reading of the high-dose MDCT by two radiologists with 4 and 6 years experience and knowledge of the fracturing protocol.

### Statistics

Inter-rater reliability is analyzed with Krippendorff's alpha [21]. A reliability from 0–0.2 is assumed to be very poor, 0.21–0.4 poor, 0.41–0.6 moderate, 0.61–0.8 good and 0.81–1 very good. Pooled sensitivity and specificity are calculated separately for fracture detection for each modality and are compared with Cochran's Q test and post hoc pairwise McNemar test. Fragment counts’ correlation to the gold standard is analyzed with Pearson's product moment correlation coefficient and compared [22]. Raters certainties are compared with Friedman test and post hoc pairwise Nemenyi test. Each rater’s fracture detection is analyzed separately, comparison between the different imaging modalities is performed with receiver operating characteristics (ROC) utilizing the DeLong method [23]. A P-value < 0.05 is assumed to denote statistical significance. Bonferroni-Holm method is applied to control the familywise error rate [24]. All confidence intervals (CI) are stated at the 95% confidence level. Because each bone is prepared and analyzed separately, statistical tests for clustering are not required. Statistical analyses are performed with R version 3.0.3.

## Results

According to the gold standard 18 out of 100 bones are fractured (see Table 1 for frequency of fractures). Inter-rater reliabilities are consistently good to moderate for RED-MDCT and RED-CBCT. We find lower values for inter-rater reliability for radiography (Table 2).

**Table 1:** Frequency of fractures.

Bone	Frequency of fractures
Radius	5
Ulna	3
Scaphoid	1
Lunate	2
Triquetrum	1
Trapezium	1
Trapezoid	1
Capitate	2
Hamate	2

**Table 2:** Inter-rater reliabilities for radiography, radiography equivalent dose multidetector CT (RED-MDCT) and radiography equivalent dose cone-beam CT (RED-CBCT) assessed with Krippendorff’s alpha.

	Radiography	RED-MDCT	RED-CBCT
fracture	0.42	0.71	0.66
fragment count	0.35	0.49	0.63

Pooled sensitivity for fracture detection is 0.53 (CI 0.40–0.67), 0.89 (CI 0.81–0.97) and 0.81 (CI 0.71–0.92) for radiography, RED-MDCT and RED-CBCT (Table 3 and S1–S3 Tables). Cochran's Q test shows significant differences between the groups (P < .001). Post hoc test reveals the sensitivity for fracture detection in RED-MDCT and RED-CBCT to be significantly higher than in radiography (P = < .004) and shows no significant difference between RED-MDCT and RED-CBCT (P = .05).

**Table 3:** Fracture sensitivity for radiography, radiography equivalent dose multidetector CT (RED-MDCT) and radiography equivalent dose cone-beam CT (RED-CBCT).

Fracture detection	Sensitivity	Sensitivity lower CI	Sensitivity upper CI	Cochrane´s Q P-value	Post hoc P-value compared to Radiography	Post hoc P-value compared to RED-MDCT
Radiography	0.54	0.40	0.67	<0.001		
RED-MDCT	0.89	0.81	0.97		<0.001	
RED-CBCT	0.81	0.71	0.92		0.004	0.05

Pooled specificity for fracture detection is 0.93 (CI 0.89–0.96), 0.93 (CI 0.90–0.96) and 0.93 (CI 0.89–0.96) for radiography, RED-MDCT and RED-CBCT (Table 4 and S1–S3 Tables). Cochran's Q test shows no significant differences between the groups (P = .98).

**Table 4:** Fracture specificity for radiography, radiography equivalent dose multidetector CT (RED-MDCT) and radiography equivalent dose cone-beam CT (RED-CBCT).

Fracture detection	Specificity	Specificity lower CI	Specificity upper CI	Cochrane´s Q P-value
Radiography	0.93	0.89	0.96	0.98
RED-MDCT	0.93	0.90	0.96	
RED-CBCT	0.93	0.89	0.96	

The fragment counts’ correlation to the gold standard of radiography 0.37 (CI 0.27–0.46), RED-MDCT 0.67 (CI 0.60–0.73) and RED-CBCT 0.50 (CI 0.41–0.58) all differ significantly (P = < .006).

**Fig 2:**
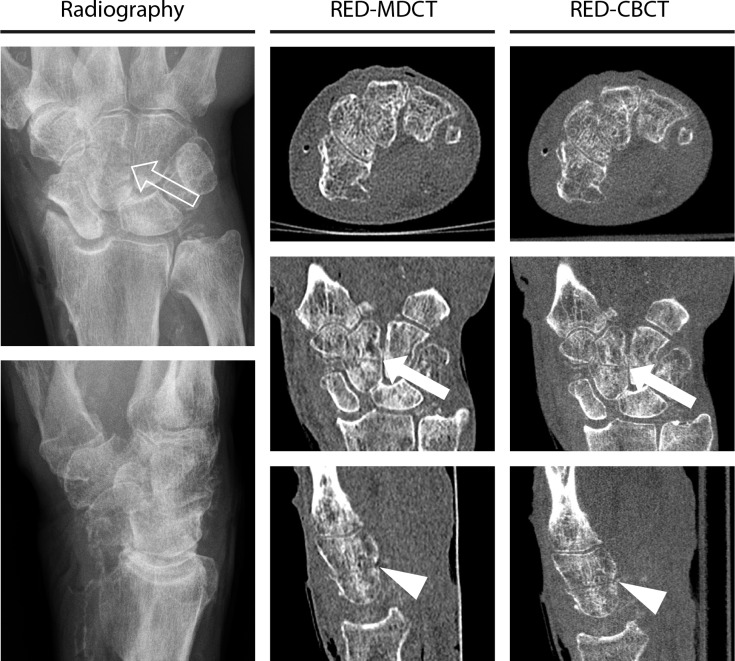
Imaging examples of one case for radiography (d.p./ lat.), radiography equivalent dose multidetector CT (RED-MDCT) and radiography equivalent dose cone-beam CT (RED-CBCT) with axial, coronal and sagittal reconstructions. The fracture of the capitate is clearly shown in the CT images (white arrows and white arrowheads), whereas radiography depicts the fracture only faintly (arrow).

**Fig 3:**
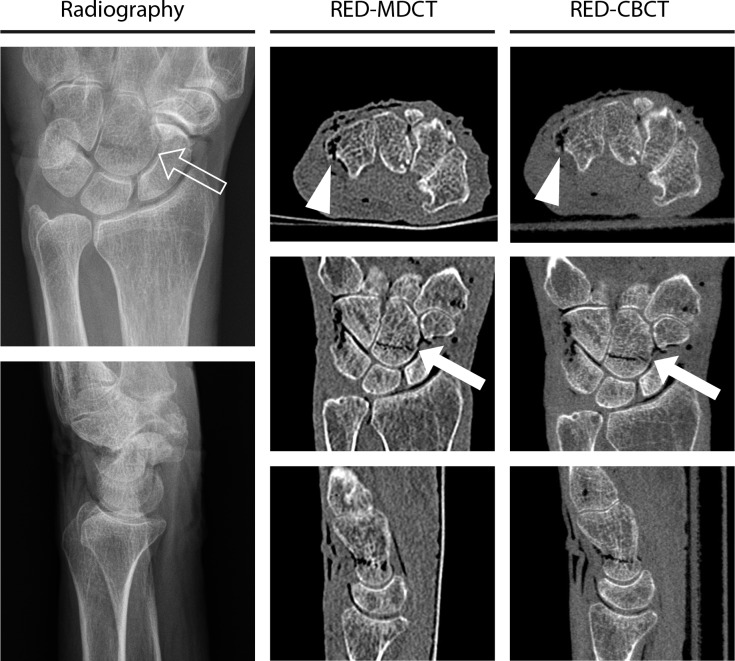
Imaging examples of one case for radiography (d.p./ lat.), radiography equivalent dose multidetector CT (RED-MDCT) and radiography equivalent dose cone-beam CT (RED-CBCT) with axial, coronal and sagittal reconstructions. The fracture of the triquetrum is only shown in the CT images (white arrowheads). The fracture of the capitate is clearly shown in the CT images (white arrows), whereas radiography depicts the fracture only faintly (arrow). In this particular case the fractures are partially filled with gas.

Friedman Test shows significant differences between raters’ certainty for fracture detection and also for fragment count in radiography, RED-MDCT and RED-CBCT (P < .001). Post hoc analysis reveals raters’ certainty for fracture detection and fragment count to be significantly higher in RED-MDCT and RED-CBCT than in radiography (P < .001). There is no significant difference regarding raters certainty for fracture detection and fragment count between RED-MDCT and RED-CBCT (P>.93). Imaging examples are given in Figs 2 and 3.

ROC-analysis for rater 1 shows an area under the curve (AUC) of 0.62, 0.92 and 0.92 for radiography, RED-MDCT and RED-CBCT. Rater 1´s AUC for RED-MDCT and RED-CBCT were significantly higher than rater 1´s AUC for radiography (P = < .004). No significant difference is detected between rater 1´s AUC for RED-MDCT and RED-CBCT.

**Fig 4:**
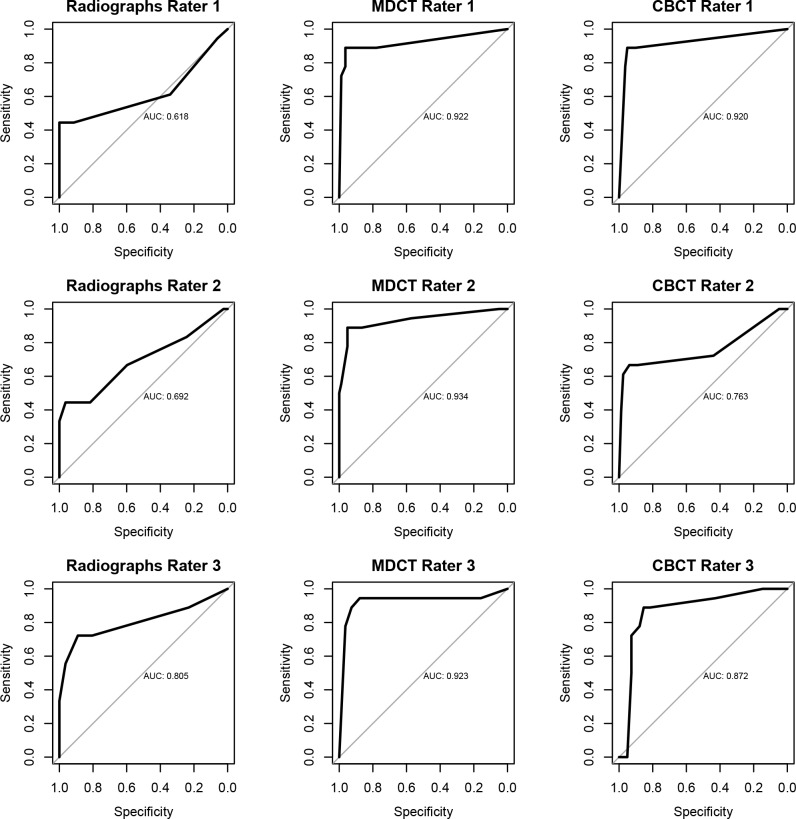
ROC-Analysis for the 3 raters regarding radiography, radiography equivalent dose multidetector CT (RED-MDCT) and radiography equivalent dose cone-beam CT (RED-CBCT).

ROC-analysis for rater 2 shows an AUC of 0.69, 0.93 and 0.76 for radiography, RED-MDCT and RED-CBCT. Rater 2´s AUC for RED-MDCT was significantly higher than rater 1´s AUC for radiography (P = < .04). No significant difference is detected between rater 1´s AUC for radiography and RED-CBCT, RED-MDCT and RED-CBCT (P = < .08). ROC-analysis for rater 3 shows an AUC of 0.81, 0.92 and 0.87 for radiography, RED-MDCT and RED-CBCT without significant differences (P>.70) (Fig 4).

## Discussion

In this study we show that the sensitivity for wrist fractures is significantly higher in RED-CT than in radiography in our experimental setting. We also demonstrate that the raters’ certainties regarding fracture detection are higher in the RED-CT compared to radiography. In addition we show the ROC analysis for fracture detection to have higher AUC-values for RED-MDCT and RED-CBCT compared to radiography, although this is significant only for two of the three raters.

The similar and in some parts even higher diagnostic accuracy for wrist fractures of RED-MDCT and RED-CBCT compared to radiography highlights radiography equivalent dose computed tomography as a potential improvement to the diagnostics of wrist fractures without raising radiation dose. Apparently, the capacity of RED-MDCT and RED-CBCT to depict bony structures without superimpositions makes it easier for the readers to detect fractures of the carpus. This most likely is also the reason why the raters´ confidence in their evaluation is significantly higher in the cross sectional imaging modalities.

Corresponding to our results, it is described in literature that radiography has a lower sensitivity for carpal fractures than the MDCT [3,4] and initial preclinical studies indicate similar results for the CBCT [25]. Regardless, a radiograph is usually carried out first in the clinical case of suspected fractures of the wrist [2]. Some authors argue that despite the increased radiation exposure early evaluation of clinically suspected fractures should be performed with CT if the radiograph appears normal [26]. This would avoid unnecessary immobilization and also prevent the delayed diagnosis of fractures. In particular, the delayed diagnosis can be a risk for complications such as delayed healing [27]. However, this strategy would also result in an increase in radiation dose. This dilemma could potentially be solved by employing cross sectional imaging without increasing the radiation dose, i.e. RED-CT scan could initially be performed instead of the radiography. Taking into account the higher sensitivity and equivalent specificity, our study suggests that the negative predictive value of RED-MDCT is higher compared to radiography. However, further studies are needed to support this assumption.

The MDCT is a well-established modality for imaging of the wrist [3,4]. In this study we performed the MDCT scan with a single shot protocol without pitch. We chose this protocol because it applies a constant radiation quantity, which sets the base for the comparison of equivalent dose examinations. This is not the case for spiral acquisition protocols, where the length of the scan and dose modulation might change the applied radiation quantity significantly in different patients. In contrast to the MDCT the CBCT is relatively new modality for the imaging of the wrist. For technical reasons, CBCT images suffer from more artifacts as compared to MDCT images [28]. In addition, iterative reconstruction and scatter correction technique were not available in our CBCT device; so the raised image noise in the low-dose study could not be suppressed in the CBCT. Thus, the increased artifacts and noise in the low-dose CBCT images of our study might hamper the diagnostic process in raters who are not accustomed to the device. This applies in particular to rater 2 (Fig 4), who had less clinical experience with the CBCT than with the MDCT.

Besides the amount of artifacts, the CBCT is also known to exceed the MDCT in terms of spatial resolution [16,17]. Nevertheless, the MDCT shows a higher fragment counts’ correlation with the gold standard than the CBCT. This result, however, is to be regarded with caution. In addition to the fracturing protocol a high-dose MDCT examination is part of the gold standard. Thus, a higher correlation of RED-MDCT with our gold standard might be explained due the same image impression particularly of minor fragments. Also pointing in this direction is the good inter-rater reliability of the RED-CBCT in this particular task. Our assumption would also be consistent with the literature, that has found no difference between CBCT and MDCT in the assessment of the fragment number [13,29].

In ROC analysis, both, RED-MDCT and RED-CBCT, significantly improve the diagnostic performance of the two less experienced readers, but not the performance of the most experienced reader. Thus, RED-MDCT and RED-CBCT might have the capacity to facilitate the diagnostic process of the wrist, i.e. improve the diagnostic accuracy even without highly experienced staff, e.g. on duties. The clinical implementation of RED-CT, however, could increase the numbers of CT scans of the wrist. To prevent that resources from a MDCT might be taken away from other urgent or emerging settings, a triage could be applied. For the CBCT this increase in numbers of scans should not be a problem as the device is dedicated to the imaging of extremities.

In our experimental study the settings of MDCT and CBCT for kVp and mAs were only stepwise adjustable. We adjusted these settings to meet the radiation dose of the radiography as close as possible without exceeding it. Thus, the resulting radiation dose of RED-MDCT and RED-CBCT lies in the same order of magnitude as the radiation dose of radiography and is actually minimally lower. Giving our results this is even more encouraging and pointing towards the high diagnostic accuracy of RED-MDCT and RED-CBCT.

Radiation exposure does preclude a study as ours to be performed in real patients–i.e. imaging the same wrist at the same time with radiography, MDCT and CBCT. Therefore we scanned formaldehyde-fixed cadaver specimens in this prospective pilot study and thus the main limitation of our study pertains to sample size. In addition, formaldehyde can demineralize bone over time and change the water and fat content of the wrist, which could hamper radiologic diagnostics, although this would apply for all three modalities tested. The artificially inflicted fractures are not fully comparable to fractures occurring in daily practice. Although we watered the specimens, a portion of the fractured bones developed emphysema (as shown in Fig 3). To a certain amount the gas will probably have been in the specimens before the fracturing process, most likely due to the handling in the anatomy department. But it is also possible, that the gas entered the specimens at the amputation site, from where it was forwarded to the bones through vessels and soft tissue. Emphysema is, however, almost never the case in patients, and could make the fracture easier to detect, especially in the cross-sectional imaging methods. We cannot exclude that the gas influenced our readers to a certain degree. Although this was less common, some non-fractured bones also contained gas and therefor gas was not a unique feature of a fracture. Hence, we do not think that the gas in the fractures relevantly changed the results regarding diagnostic accuracy, which was the aim of our study. Because the decision to fracture the bones was taken at random (dice roll), we were not able to integrate fracture patterns. Also, the resulting frequency of the fractures in this study is very unlikely to occur in clinical practice. The results are limited to the imaging protocols and devices used in this study, i.e. a 320 row MDCT with single shot mode and a CBCT dedicated to musculoskeletal imaging.

To sum up we demonstrate that the diagnostic accuracy of RED-MDCT and RED-CBCT for wrist fractures is similar and in some parts even higher compared to radiography. Readers are more confident in their reporting with the cross sectional modalities. Our findings suggest that dose equivalent cross sectional computed tomography imaging of the wrist could replace plain radiography for fracture diagnosis in the long run. Further clinical studies should be performed to validate these results.

## Supporting Information

S1 Table Contingency table for Radiography. (DOCX)

S2 Table Contingency table for Radiography equivalent dose multidetector computed tomography (RED-MDCT). (DOCX)

S3 Table Contingency table for Radiography equivalent dose cone beam computed tomography (RED-CBCT). (DOCX)

S4 Table Raw data with ratings and gold standard. (TXT)
