# Supplementary material for: Comparison of Diagnostic Accuracy of Radiation Dose-Equivalent Radiography, Multidetector Computed Tomography and Cone Beam Computed Tomography for Fractures of Adult Cadaveric Wrists
Source: PLoS One. 2016 Oct 27;11(10):e0164859. doi: 10.1371/journal.pone.0164859 (PMC5082876; doi:10.1371/journal.pone.0164859)
Supplement: S2 Table — (DOCX) [file pone.0164859.s002.docx]

| RED-MDCT | Fracture | Intact |
| --- | --- | --- |
| RED-MDCT positive | 48 | 17 |
| RED-MDCT negative | 6 | 229 |
